# Function of Lipid Storage Droplet 1 (Lsd1) in Wing Development of *Drosophila melanogaster*

**DOI:** 10.3390/ijms17050648

**Published:** 2016-04-29

**Authors:** Tran Thanh Men, Tran Duy Binh, Masamitsu Yamaguchi, Nguyen Tien Huy, Kaeko Kamei

**Affiliations:** 1 Department of Biomolecular Engineering, Kyoto Institute of Technology, Kyoto 606-8585, Japan; ttmen@ctu.edu.vn (T.T.M.); tdbinh22@gmail.com (T.D.B.); 2 Department of Applied Biology, Kyoto Institute of Technology, Kyoto 606-8585, Japan; myamaguc@kit.ac.jp; 3 Department of Clinical Product Development, Institute of Tropical Medicine (NEKKEN), Nagasaki University, Nagasaki 852-8523, Japan; tienhuy@nagasaki-u.ac.jp

**Keywords:** *Drosophila*, lipid storage droplet 1, mitochondrial stress, perilipin, wing development

## Abstract

Perilipins are evolutionarily conserved from *Drosophila* to humans, the lipid storage droplet 1 (*Lsd1*) is a *Drosophila* homolog of human perilipin 1. The function of *Lsd1* as a regulator of lipolysis in *Drosophila* has been demonstrated, as the *Lsd1* mutant causes an increase of lipid droplet size. However, the functions of this gene during development are still under investigation. In order to determine the function of *Lsd1* during development, *Lsd1* was knocked down in *Drosophila* using the GAL4-UAS system. Selective knockdown of *Lsd1* in the dorsal wing disc caused an atrophied wing phenotype. The generation of reactive oxygen species in the wing pouch compartment of the *Lsd1*-knockdown flies was significantly higher than in the control. Immunostaining with caspase-3 antibody revealed a greater number of apoptotic cells in *Lsd1*-knockdown wing discs than in the control. Cell death by autophagy was also induced in the knockdown flies. Moreover, cells deprived of *Lsd1* showed mitochondrial expansion and decreased ATP levels. These results strongly suggest that knockdown of *Lsd1* induces mitochondrial stress and the production of reactive oxygen species that result in cell death, via apoptosis and the autophagy pathway. These results highlight the roles of *Drosophila* *Lsd1* during wing development.

## 1. Introduction

Triacylglycerols (TAG) in adipose tissue are the body’s major energy storage source [1]. Lipid storage organelles, known as lipid droplets (LDs), are abundant in the adipose tissue and play a role in controlling the body fat banks of animals [2]. LDs are composed of a neutral lipid core coated by a lipid monolayer with proteins, the best known of which is a protein family named PAT domain proteins. PAT domain proteins include ADRP and TIP47, and are collectively named perilipins [3]. The interaction between perilipins and lipases in LDs is related to the regulation of lipid homeostasis. Moreover, perilipin 1 is the most well-characterized member of the PAT family, and regulates basal and stimulated lipolysis in opposite ways. In normal conditions, perilipin 1 blocks the access of lipases to LDs and suppresses adipose triglyceride lipase (ATGL) activation by forming a complex with CGI-58, an activator of ATGL. In times of energy deficit, perilipin 1 is phosphorylated by protein kinase A (PKA) in response to hormonal signals, and phosphorylated perilipin 1 facilitates maximal lipolysis by recruiting hormone-sensitive lipase (HSL) and allowing ATGL to access the LD [4,5,6,7]. The mutation of perilipin 1 in mice results in a lean phenotype, and a lack of perilipin 1 combined with a mutation in the leptin receptor gene in mice reverses obesity. These phenotypes are derived from loss of the anti-lipase protective effect of perilipin 1 under normal conditions [8]. In humans, genetic variations in the perilipin 1 gene have been associated with metabolic disorders, including type 2 diabetes and partial lipodystrophy [9,10]. In addition to perilipin 1, there are four other mammalian perilipins: ADRP/perilipin 2, TIP47/perilipin 3, S3-12/perilipin 4, and OXPAT/perilipin 5 [9]. Perilipins are evolutionarily conserved from *Drosophila* to humans. *Drosophila* has only two perilipins, perilipin 1/Lipid storage droplet 1 (Lsd1), and perilipin 2/Lipid storage droplet 2 (Lsd2) [9]. More importantly, *Drosophila* and higher animals share the same basic metabolic functions and lipid metabolism-related genes [11,12]. The function of *Lsd1* in lipid metabolism in *Drosophila* is well known. For example, analyses with GFP (green fluorescent protein)-tagged *Lsd1* displayed its presence on the surface of LDs in *Drosophila* fat body cells [13]. In addition, loss of function or overexpression of *Lsd1* in *Drosophila* indicated that *Lsd1* probably facilitates lipid mobilization [8]. *In vitro* studies identified *Lsd1* as a PKA phosphorylation target [14], while *in vivo* mutant analysis demonstrated an essential role of *Lsd1* as a pro-lipolytic effector of the AKH/AKHR pathway on the LD surface [2]. To date, other functions and genetic regulatory mechanisms of this gene are still under investigation.

In this study, the function of *Lsd1* was further investigated in *Drosophila* by selective knockdown of the *Lsd1* gene using the GAL4-UAS targeted expression system in combination with RNA interference [15]. By crossing tissue and developmentally specific GAL4 driver fly lines with a fly line carrying the UAS-*Lsd1*IR (IR, inverted repeat), the *Lsd1* gene can be specifically knocked down in any desired tissue or developmental stage. The knockdown experiments in this study revealed that *Lsd1* is necessary for the development of *Drosophila* wings, possibly through maintaining the function of mitochondria.

## 2. Results

### 2.1. Effect of Lsd1 Knockdown in Various Tissues and Entire Drosophila

We knocked down *Lsd1* by crossing the UAS-*Lsd*1I*R* fly line with several GAL4 driver lines. As summarized in Table 1, *Lsd1* knockdown in the whole fly by Act5C-GAL4 or Tubp-GAL4 resulted in a lethal phenotype at the embryonic stage. *Lsd1* knockdown by En-Gal4 also caused lethality, probably because of the reported leaky expression of GAL4 during embryogenesis [16,17]. These results indicate an essential role of the *Lsd1* gene for viability and/or development of *Drosophila. Lsd1* knockdown in the fat body caused a delay in growth at 25 °C and lethality at 28 °C. These results are consistent with previous studies of *Lsd1* mutants and indicate that *Lsd1* plays an important role in lipid metabolism [8]. The specific knockdown of the *Lsd1* gene in wing discs with MS1096-GAL4 driver resulted in a severe atrophied wing phenotype, suggesting that *Lsd1* plays unexplored role/s during wing development. Eye disc-specific knockdown of the *Lsd1* gene by GMR-GAL4 (at 28 °C) exhibited no detectable phenotype, suggesting that *Lsd1* plays no apparent role during eye development. These observations suggest the tissue-specific role of *Lsd1* in the development, although the possibility of low level expression of GAL4 protein leading to insufficient knockdown of *Lsd1* in eye disc can not be excluded.

### 2.2. Knockdown of Lsd1 Disrupted Normal Wing Development

**Figure 1:**
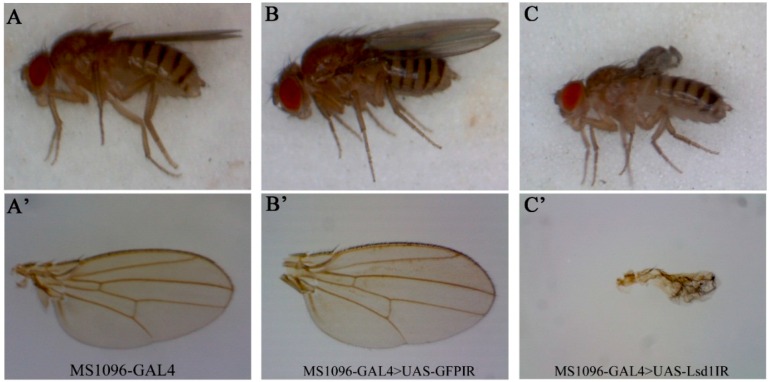
Morphological aberrations in the adult wing of *Lsd1*-knockdown flies by MS1096-GAL4 driver. (**A**–**C**) whole body of adult flies; (**A’**–**C’**) enlarged images of adult wings. (**A**,**A’**) control fly (MS1096-GAL4/+; +; + (MS1096-GAL4)); (**B**,**B’**) control fly carrying *GFP*IR gene (MS1096-GAL4/+; UAS-*GFP*IR/+; + (MS1096-GAL4 > UAS-*GFP*IR)); (**C**,**C’**) *Lsd1*-knockdown fly (MS1096-GAL4/+; UAS-*Lsd1*IR/+; + (MS1096-GAL4 > UAS-*Lsd1*IR)). Flies were reared at 25 °C. Control flies, MS1096-GAL4 (**A**,**A’**) and MS1096-GAL4 > UAS-*GFP*IR (**B**,**B’**) showed normal wing phenotypes, while *Lsd1*-knockdown fly had atrophied wing (**C**,**C’**).

Although the function of *Lsd1* in lipid metabolism is well known, its potential function in wing development has not been explored. We therefore focused on the analyses of the wing phenotype induced by *Lsd1* knockdown. Flies carrying a single copy of the MS1096-GAL4 driver and UAS-*Lsd1*IR (MS1096-GAL4 > UAS-*Lsd1*IR) exhibited severe atrophied wing phenotypes, compared to control flies carrying one copy of MS1096-GAL4 only or flies carrying both MS1096-GAL4 and UAS-*GFP*IR (MS1096-GAL4 > UAS-*GFP*IR) (Figure 1). This effect may be caused by the disruption of the developmental processes of the wings.

**Figure 2:**
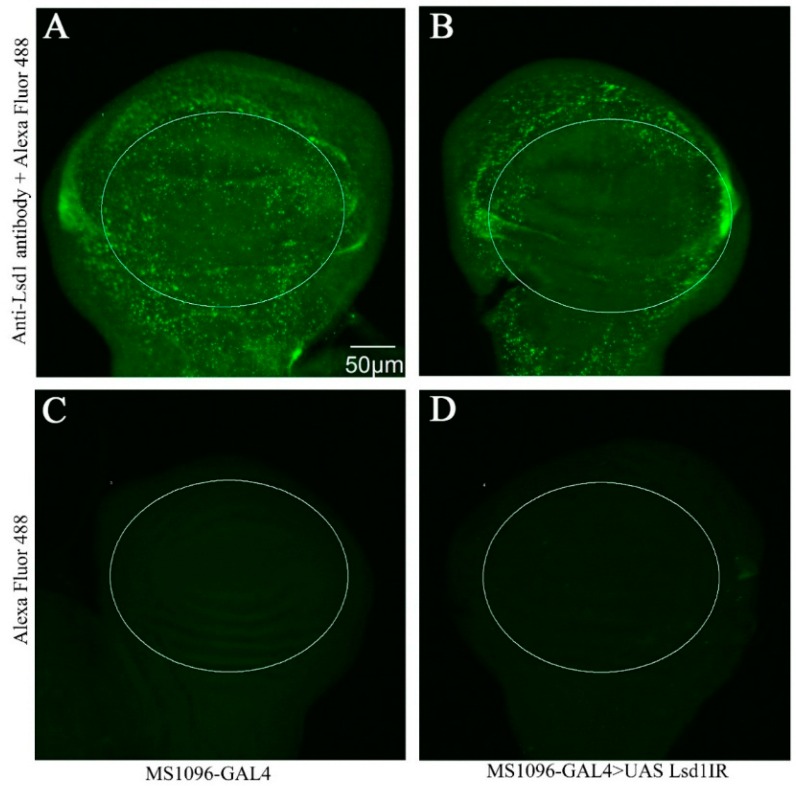
Immunostaining of wing imaginal discs with anti-Lsd1 antibody. (**A**,**B**) wing imaginal discs were reacted with rabbit anti-Lsd1 antibody followed by anti-rabbit IgG Alexa Fluor™ 488 antibody; MS1096-GAL4 flies (**A**) showed signal clearly and MS1096-GAL4 > UAS-*Lsd1*IR flies (**B**) showed decreased Lsd1 signal in the wing pouch of wing imaginal discs (**C**,**D**) immunostaining of wing imaginal discs with only the anti-rabbit IgG Alexa Fluor^TM^ 488 antibody showed no detectable signal; (**C**) MS1096-GAL4; (**D**) MS1096-GAL4 > UAS-*Lsd1*IR. Flies were reared at 25 °C. The circles indicate wing pouch of wing discs where *Lsd1* was knocked down.

To confirm the effective knockdown of *Lsd1* in the dorsal wing disc, we performed immunostaining of wing imaginal discs from third instar larvae using anti-Lsd1 antibody. The specificity of the anti-Lsd1 antibody we used has been fully characterized [2]. *Lsd1*-knockdown flies (MS1096-GAL4 > UAS-*Lsd1*IR) showed an extensive decrease of Lsd1 signals in the wing pouch of the wing imaginal disc (circled in Figure 2B). Control flies carrying MS1096-GAL4 alone showed strong Lsd1 signals in the wing pouch as well as other regions of the wing disc (Figure 2A). Immunostaining of wing imaginal discs of both MS1096-GAL4 and MS1096-GAL4 > UAS-*Lsd1*IR flies using only the secondary antibody showed no detectable signal in wing discs (Figure 2C,D). These results confirmed the effective knockdown of *Lsd1* in the wing disc of MS1096-GAL4 > UAS-*Lsd1*IR flies, which very likely resulted in the atrophied wing phenotype.

### 2.3. Knockdown of Lsd1 Led to Increased Cell Death

**Figure 3:**
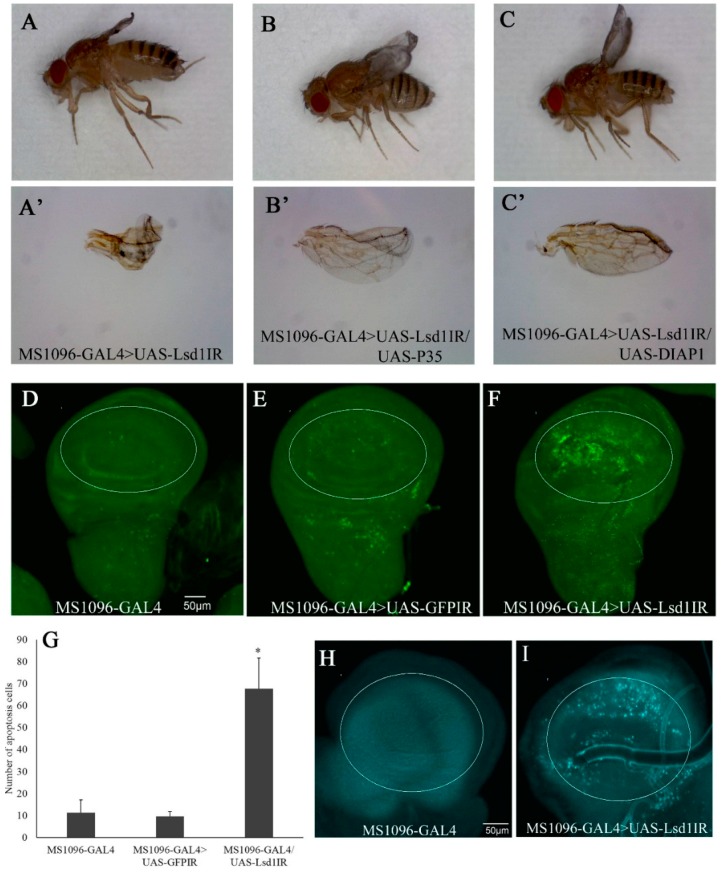
Knockdown of *Lsd1* induces cell death in wing imaginal discs. The atrophied wing phenotype of *Lsd1*-knockdown flies was rescued by P35 (**B**,**B’**) and DIAP1 (**C**,**C’**); (**A**,**A’**) MS1096-GAL4 > UAS-*Lsd1*IR; (**B**,**B’**) MS1096-GAL4/+; UAS-*Lsd1*IR/+; UAS-P35/+ (MS1096-GAL4 > UAS-*Lsd1*IR/UAS-P35); (**C**,**C’**) MS1096-GAL4/+; UAS-*Lsd1*IR/+; UAS-DIAP1/+ (MS1096-GAL4 > UAS-*Lsd1*IR/UAS-DIAP1); (**D**–**F**) immunostaining of wing imaginal discs with anti-active caspase-3 antibody; Control lines, MS1096-GAL4 (**D**) and MS1096-GAL4 > UAS-*GFP*IR (**E**); showed few cell death signals, while *Lsd1*-knockdown line MS1096-GAL4 > UAS-*Lsd1*IR (**F**) showed increased cell death signals via apoptosis; (**G**) average number of apoptotic cells in the wing pouch (*n* = 10); *, *p* < 0.05. Data are expressed as the mean ± S.D; (**H**,**I**) autophagy was determined by LysoTracker staining; (**H**) the control fly MS1096-GAL4; (**I**) *Lsd1*-knockdown fly MS1096-GAL4 > UAS-*Lsd1*IR. The circle indicates the wing pouch of the wing disc. The flies were reared at 25 °C.

The atrophied wings of *Lsd1*-knockdown flies suggested the possible involvement of apoptotic and/or autophagy processes. In *Drosophila*, baculoviral P35 and DIAP1 (Death-associated inhibitor of apoptosis 1) have been shown to effectively inhibit apoptosis when ectopically expressed [18]. *Lsd1-*knockdown flies were crossed with the flies carrying UAS-P35 or UAS-DIAP1. The atrophied wing phenotype induced by *Lsd1-*knockdown (Figure 3A,A’) was effectively suppressed by wing specific expression of P35 (Figure 3B,B’) or DIAP1 (Figure 3C,C’). These data suggest that atrophied wing phenotype induced by *Lsd1-*knockdown is at least partially due to apoptosis in wing imaginal discs. Therefore, wing discs from third instar larvae of *Lsd1*-knockdown and control flies were immunostained with anti-cleaved caspase-3 IgG to detect caspase-dependent apoptotic cells. The wing pouch compartment of the wing imaginal disc of *Lsd1-*knockdown flies (MS1096-GAL4 > UAS-*Lsd1*IR) showed an increase in apoptotic cells compared with the control (Figure 3D–F). As shown in Figure 3G, the difference in the number of apoptotic cells in the wing pouches of *Lsd1-*knockdown flies and control flies was statistically significant (*p* < 0.05, Student’s *t* test). These results indicate that knockdown of *Lsd1* in the wing disc induces apoptosis.

We also examined autophagy in *Lsd1*-knockdown flies using LysoTracker Blue, which showed that, compared with the control, *Lsd1*-knockdown induced autophagy (Figure 3H,I). Since it is reported that over production of reactive oxygen species (ROS) causes both apoptosis [19] and autophagy [20], these data suggest that ROS generation may accumulate in dorsal wing discs of *Lsd1*-knockdown flies and cause cell death via apoptotic and autophagy pathways.

### 2.4. Knockdown of Lsd1 Increased ROS Generation

**Figure 4:**
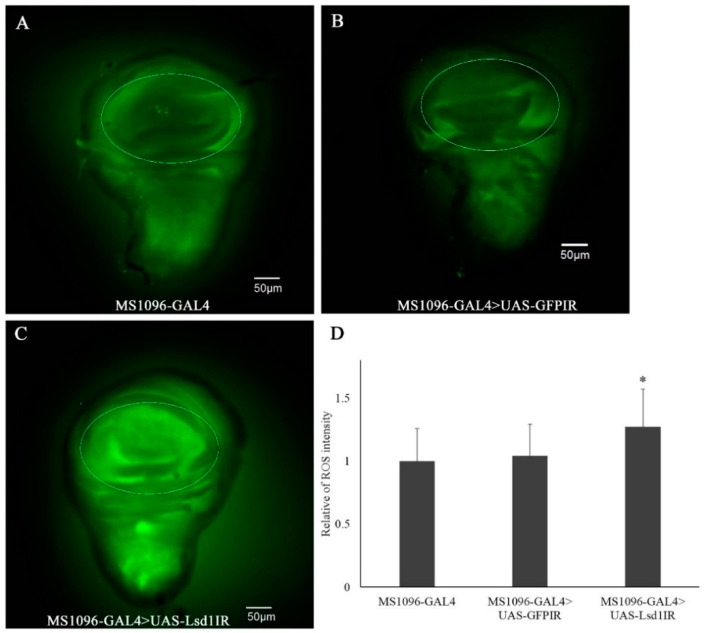
Knockdown of *Lsd1* induces ectopic ROS in wing imaginal discs. Wing discs of third-instar larvae were incubated with substrate CM-H_2_DCFDA. The control lines MS1096-GAL4 (**A**) and MS1096-GAL4 > UAS-*GFP*IR (**B**) showed an unclear ROS signal in the wing disc (marked by the circle), while the *Lsd1*-knockdown line (MS1096-GAL4 > UAS-*Lsd1*IR) showed an increased ROS signal (**C**); (**D**) Quantification of fluorescent ROS signals in the wing pouch region. Mean intensities with standard deviation from six imaginal discs are shown; * *p* < 0.05, Student’s *t* test.

To detect intracellular ROS in *Lsd1*-knockdown flies, we used the non-fluorescent substrate CM-H_2_DCFDA, which can be oxidized by ROS to an intracellular green fluorescent product. Faint signals were detected in wing imaginal discs of MS1096-GAL4 and MS1096-GAL4 > UAS-*GFP*IR control flies as shown in Figure 4A,B, respectively. In contrast, much stronger fluorescent signals were detected in the wing pouch region of imaginal discs from *Lsd1*-knockdown flies (Figure 4C). The ROS signal detected outside of the wing pouch in Figure 4C could be explained by the non-cell autonomous effect. The quantified data of fluorescent signals (Figure 4D) confirmed that ROS generation was induced by the knockdown of *Lsd1* in wing discs.

### 2.5. Knockdown of Lsd1 Caused Stress in Mitochondria and Defects in ATP Production

**Figure 5:**
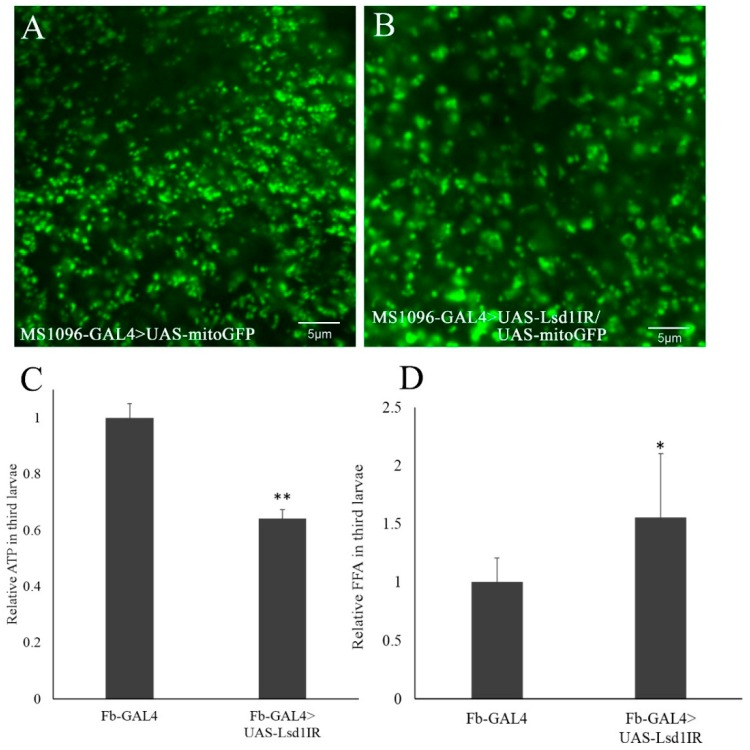
Knockdown of *Lsd1* induces mitochondrial expansion and defects in ATP and free fatty acids (FFA) production. (**A**,**B**) The green fluorescent protein (GFP) signal expressed in mitochondria of wing disc cells were observed under BX-50 fluorescence microscopy. The control flies MS1096-GAL4/+; +; UAS-MitoGFP/+ (MS1096-GAL4 > UAS-MitoGFP) showed normal mitochondrial matrix (**A**); while the *Lsd1*-knockdown flies MS1096-GAL4/+; UAS-*Lsd1*IR/+; UAS-MitoGFP/+ (MS1096-GAL4 > UAS-*Lsd1*IR/UAS-MitoGFP) appeared to exhibit mitochondrial expansion (**B**); Relative amounts of ATP (**C**) and FFA (**D**) of *Lsd1*-knockdown flies *yw*; Fb-GAL4/UAS-*Lsd1*IR; + (Fb-GAL4 > UAS-*Lsd1*IR). ATP and FFA amounts in whole bodies of third instar larvae were normalized to body weight. The experiments were repeated at least three times. * *p* < 0.05, ** *p* < 0.001, Student’s *t* test. The flies were reared at 25 °C.

The results described above suggest the primary effect of *Lsd1* knockdown during *Drosophila* wing development is ROS production, followed by induction of apoptosis and autophagy. A number of studies have demonstrated that mitochondria are an important source of ROS within cells [21,22,23]. Therefore, we examined whether the *Lsd1*-knockdown could affect mitochondrial function in *Drosophila*. First, we examined the morphology of mitochondria in *Lsd1*-knockdown flies by simultaneously expressing UAS-MitoGFP. MitoGFP has been widely used to mark mitochondria in both *Drosophila* and mammalian cells by expressing GFP fusion proteins containing the mitochondrial-targeting sequence of citrate synthase [24,25,26]. Compared to control flies (Figure 5A), the mitochondrial morphology in wing imaginal discs appeared to be expanded in *Lsd1*-knockdown flies expressing MitoGFP (MS1096-GAL4 > UAS-*Lsd1*IR/UAS-MitoGFP) (Figure 5B). These data suggest that mitochondria are under stress in the *Lsd1*-knockdown flies.

Mitochondria are responsible for the production of ATP, a major cellular energy source [27,28]. Since fat body in *Drosophila* is known for its importance not only for energy storage but also lipid metabolism, similarly to the mammalian liver [29], we measured the amount of ATP in entire third instar larvae in which *Lsd1* was knocked down in the fat body by the Fb-GAL4 driver. Compared to control flies carrying Fb-GAL4 alone, the ATP level in *Lsd1*-knockdown flies (Fb-GAL4 > UAS-*Lsd1*IR) was decreased (Figure 5C). Moreover, accumulation of free fatty acids may result in increased fatty acid uptake and oxidation and may ultimately increase ROS production [30]. We then examined total free fatty acids (FFA) levels, and found that FFA levels were significantly elevated in *Lsd1*-knockdown flies compared with the control (Figure 5D). These data strongly suggest a defect in mitochondrial function caused by *Lsd1*-knockdown.

## 3. Discussion

*Lsd1* has been reported to act as a conserved surface-associated module of lipid droplets that promotes stimulated lipolysis by response to cAMP/PKA signaling [2]. PKA-dependent perilipin phosphorylation and recruitment of HSL to LDs, which are distinctive features of stimulated lipolysis in mammalian adipocytes, led to the discovery of a conserved function of the TG mobilization module between flies and mammals [31]. However, to date, there has been no report of other possible *Lsd1* functions during development. This study demonstrates that knockdown of the *Lsd1* gene in the entire *Drosophila melanogaster* body caused lethality, indicating that *Lsd1* is an essential function for viability. When *Lsd1* was knocked down in wing discs using the MS1096-GAL4 driver, severely atrophied wings were observed. Cell death in the wing pouch of *Lsd1* knockdown flies was demonstrated to be due to an increase in apoptosis and autophagy. Previous reports have shown that the increase in ROS production is accompanied by apoptosis in eukaryotic cells [32,33]. Our observations demonstrated that knockdown of *Lsd1* expressed a causal signal of increasing ROS generation, which might be related to apoptosis and autophagy in wing imaginal discs. Further analysis is necessary to clarify this point. Moreover, the atrophied phenotype of *Lsd1*-knockdown flies in the present study was rescued by the expression of anti-apoptotic genes DIAP1 and P35 [18]. Overall, these results indicated that increased ROS generation might cause apoptosis and autophagy and result in the atrophied wing phenotype in adults.

Perilipin 1 has a central role in modulating adipocyte lipid metabolism. Under normal conditions, perilipin 1 suppresses maximal lipolysis by forming a complex with CGI-58/abhydrolase domain-containing protein 5 (ABHD5), which is an activator of Brummer (BMM) in *Drosophila* (corresponding to ATGL) [2]. Interestingly, the interaction between CGI-58 and perilipin 1 is sensitive to hormonal stimulation. The lipolytic activation of fat cells disrupts this interaction and leads to the dissociation of CGI-58 from the complex, resulting in the binding of CGI-58 and ATGL for maximal lipolysis [34]. We hypothesize that absence of *Lsd1* may lead to the release CGI-58 and enhance the activity of BMM. Enhanced BMM activity can promote the increase of free fatty acids, which enter mitochondria and are responsible for ATP synthesis. Specifically, the overproduction of free fatty acids can cause mitochondrial stress, increase ROS production, and lead to cell death [35]. Mitochondrial stress can also cause a decrease of ATP levels and affect the mitochondrial morphology [36]. In the present study, we observed that the morphology of mitochondria appeared to be expanded in wing imaginal discs, and the ATP level decreased in *Lsd1-*knockdown flies. In addition, the free fatty acids level in knockdown flies was also significantly elevated. Taken together, these observations at least partially support our hypothesis that increased free fatty acids in *Lsd1*-knockdown flies cause mitochondrial stress and consequently induces cell death by the apoptotic and autophagy pathways, thereby ultimately affecting wing phenotype.

## 4. Materials and Methods

### 4.1. Fly Stocks

Fly stocks were maintained at 25 °C on standard food (4% dry yeast, 9% cornmeal, 10% glucose, 0.8% agar, 0.5% propionic acid, and 0.05% ethyl parahydroxybenzoate). RNAi stock v30884 (FBgn0039114) with no off target effects, and carrying inverted repeats of the *Lsd1* gene (targeting the region from nucleotide number 437–782 of *Lsd1* mRNA) located on the second chromosome (*w*; UAS-*Lsd1*IR; +), was obtained from the Vienna *Drosophila* Resource Center (VDRC, Vienna, Austria). The FB-GAL4 driver line (expressed in the fat body) was kindly supplied by Ronald P. Kuhnlein of the Max Planck Institute for Biophysical Chemistry (Göttingen, Germany) [37]. All other lines used in this study were obtained from the Bloomington Stock Center at Indiana University (Bloomington, IN, USA).

The UAS-*Lsd*1I*R* fly line was crossed with several GAL4 drivers to knockdown *Lsd1* in specific tissues of the F1 generation. For dorsal wing disc-specific knockdown of *Lsd1*, virgin female MS1096-GAL4 flies, with two copies of the MS1096-GAL4 gene on the X chromosome (MS1096-GAL4; +; +), were crossed with male UAS-*Lsd1*IR flies with two copies of the UAS-*Lsd1*IR gene on the second chromosome (*w*; UAS-*Lsd1*IR; +). Yellow white (*yw*) and the *Drosophila* carrying inverted repeats of green fluorescent protein gene (*yw*; UAS-*GFP*IR; +) were crossed with the female MS1096-GAL4 driver as controls. *Lsd1* was knocked down in the posterior wing disc and embryo (using en-GAL4 driver), in eye discs (using GMR-GAL4 driver), in the fat body (using Fb-GAL4 driver), and ubiquitously throughout tissues (using Act5C-GAL4 driver or Tubp-GAL4 driver). All knockdown experiments were performed by crossing UAS-*Lsd1*IR flies with the specific driver line.

### 4.2. Immunohistochemistry

Third instar larvae were dissected in phosphate buffered saline (PBS), and wing discs were fixed in 4% paraformaldehyde for 30 min at 25 °C. After washing with 0.3% Triton X-100 in PBS (PBST), samples were blocked for 30 min at 25 °C with 0.15% PBST containing 1% bovine serum albumin. Samples were then incubated with rabbit anti-Lsd1 antibody (kindly provided by Ronald P. Kuhnlein of the Max Planck Institute for Biophysical Chemistry, Germany) [2] at a 1:1000 dilution, at 4 °C for 16 h. Following an extensive wash with PBST, the samples were incubated with goat anti-rabbit IgG Alexa Fluor™ 488 (Molecular Probes, Invitrogen, Rockford, IL, USA) at a 1:400 dilution for 2 h at 25 °C, further washed with PBST and PBS, and mounted in Vectashield mounting medium (Vector Laboratories, Tokyo, Japan). Samples were inspected with a fluorescence BX-50 microscope (Olympus, Tokyo, Japan) equipped with a cooled CCD camera (ORCA-ER; Hamamatsu Photonics K.K., Shizuoka, Japan).

### 4.3. In Vivo ROS Detection

Wing discs were collected from the third instar larvae in PBS and then incubated with 10 μM CM-H_2_DCFDA (5-(and-6)-carboxy-2′,7′-dichlorodihydrofluorescein diacetate, acetyl ester) (Molecular Probes, Invitrogen) for 5 min to detect ROS. After washing with PBS, samples were fixed in 1% paraformaldehyde for 5 min, washed three times with PBS, and then mounted in Vectashield mounting medium. Preparations were examined under a fluorescence BX-50 microscope equipped with a cooled CCD camera. The fluorescence intensity in the wing pouch was analyzed using MetaMorph software (version 7.7.7.0; Molecular Devices, Sunnyvale, CA, USA), and then subtracted by that of outside of wing disc.

### 4.4. Apoptotic Detection

Third instar larvae were dissected in PBS, and wing discs were fixed in 4% (*w*/*v*) paraformaldehyde in PBS for 30 min at 25 °C. After washing with PBST, samples were blocked for 30 min at 25 °C with 0.15% (*v*/*v*) Triton X-100 in PBS containing 1% (*w*/*v*) bovine serum albumin. Samples were then incubated with rabbit anti-cleaved caspase-3 IgG (Cell Signaling Technology, Tokyo, Japan) at a 1:100 dilution for 16 h at 4 °C. After extensive washing with PBST, the samples were incubated with goat anti-rabbit IgG Alexa Fluor™ 488 (Molecular Probes, Invitrogen) at a 1:400 dilution for 2 h at 25 °C, washed with PBST and PBS, and then mounted in Vectashield mounting medium. Preparations were examined under a fluorescence BX-50 microscope equipped with a cooled CCD camera. Apoptotic cells were counted using MetaMorph software.

### 4.5. Autophagy Assay

Larval tissues dissected in PBS were subjected to the process of acidic organelle staining, including autolysosome. Samples were incubated with 100 nM LysoTracker Blue (Invitrogen) in PBS for 2 min, washed twice in PBS, and mounted in 50% glycerol in PBS. Preparations were immediately examined by a fluorescence BX-50 microscope to obtain images.

### 4.6. ATP Assay

We used CellTiter-Glo^®^ luminescent cell viability assay kit (Promega, Madison, WI, USA) to quantify ATP. Assay kit buffer (100 μL) was used for homogenizing each adult fly, and the homogenate was centrifuged at 12,000× *g* for 10 min. The supernatant was collected, and 10 μL of the supernatant was mixed with 100 μL of measure buffer. Luminescent signals were maintained by incubating the compound at 25 °C for 10 min before being read on a Lumat LB 9507 luminometer (Berthold, Bad Wildbad, Germany).

### 4.7. FFA Level Measurement

Free fatty acid (FFA) levels were measured using a free fatty acid quantification kit from Sigma (catalog number MAK044-1KT, Tokyo, Japan) with the procedure as described in the instruction manual. Briefly, five third instar larvae were homogenized in 200 μL of 1% Triton X-100-chloroform, and the debris was removed by centrifugation. The organic phase (50 μL) was collected, dried by N_2_ gas flush, 200 μL of fatty acid assay buffer was added (from the assay Kit, Tokyo, Japan) and then dissolved by extensive vortex mixing for 5 min. For FFA measurement, 50 μL of extracted sample was used. The relative FFA level of flies was calculated against those of control flies. All measurements were performed at least three times.

## 5. Conclusions

In this study, we present data which indicate that knockdown of *Lsd1* could induce mitochondrial stress and result in cell death via apoptotic and autophagy pathways. Therefore, we conclude that *Lsd1* is necessary for the development of *Drosophila* wings, possibly through maintaining the function of mitochondria. This is the first report stating the role of *Lsd1* during *Drosophila* development and will provide the starting point for further elucidation of the roles of *Lsd1* in *Drosophila* development.

## Figures and Tables

**Table 1:** Summary of phenotypes induced by knockdown of *Lsd1* with various GAL4 driver lines.

GAL4 Line	Expression Pattern	Phenotype with UAS-*Lsd1*IR
MS1096	Dorsal wing disc	Atrophied wing at 25 and 28 °C
En	Posterior wing disc and embryo	Lethal at 25 °C
GMR	Eye disc	No detectable phenotype
Fb	Fat body	Delay in growth at 25 °C, lethal at 28 °C
Act5C	All tissues	Lethal at 25 °C
Tubp	All tissues	Lethal at 25 °C
